# Supplementary material for: Cognitive Performance and Health-Related Quality of Life in Patients with Neuromyelitis Optica Spectrum Disorder
Source: J Pers Med. 2022 May 2;12(5):743. doi: 10.3390/jpm12050743 (PMC9146457; doi:10.3390/jpm12050743)
Supplement: Supplementary file 1 [file jpm-12-00743-s001.zip › jpm-1633004-supplementary.pdf]

Supplementary material

Table S1. Demographic, clinical and patient-centred measures differences between AQP4-IgG-positive and -negative patients.

	AQP4-IgG positive (*n* = 27)	AQP4-IgG negative (*n* = 13)	Corrected *p*-value
**Demographic and clinical data**			
Age (years)	46 (39.5 – 49)	42 (28 – 47)	0.464[b]
Female, n (%)	25 (93)	9 (69)	0.428[a]
Disease duration (years)	8.3 (6.3 – 17.6)	4.8 (1.6 – 10.1)	0.204[b]
EDSS score (range)	2.0 (1.0 – 7.5)	2.0 (0 – 6.0)	0.942[b]
Number of relapses	2.5 (1 – 4)	3 (2 – 3.5)	0.598[b]
Current treatment, n (%)	27 (100)	9 (69)	0.120[a]
Beck Depression Inventory-Fast Screen (BDI-FS)	3 (0 – 7.5)	3 (2 – 5)	0.570[b]
Fatigue Impact Scale for Daily Use (D-FIS)	6 (2 – 18.5)	12 (3 – 17)	0.598[b]
**Patient-centred measures**			
Physical MSIS-29v2	33 (23 – 48.5)	40 (28 – 59)	0.915[c]
Psychological MSIS-29v2	18 (12.5 – 26)	25 (17 – 33)	0.620[c]
Satisfaction with Life Scale (SWLS)	21 (18 – 28)	21 (18 – 24)	0.915[c]
SymptoMScreen questionnaire (SyMS)	19 (7 – 29.5)	16 (10 – 32)	0.915[c]
Stigma Scale for Chronic Illness (SSCI-8)	9 (8 – 13)	12 (9 – 15)	0.620[c]
MOS Pain Effects Scale (PES)	14 (9 – 19.5)	17 (9 – 20)	0.914[c]

The data represent the absolute numbers and proportions of qualitative data and the median and IQR for quantitative data, unless otherwise specified. AQP4-IgG: aquaporin-4 immunoglobulin G; EDSS: Expanded Disability Status Scale; MSIS-29v2: Multiple Sclerosis Impact Scale. One patient was excluded due to unknown serostatus.

[a]Chi-squared test; [b]Kruskal–Wallis test; [c]Age and sex-adjusted ANOVA and corrected by FDR adjustment.

Table S2. Demographic, clinical and patient-centred measures differences between CI and CP patients.

	CI patients (n = 14)	CP patients (n = 27)	Corrected p-value
**Demographic and clinical data**			
Age (years)	45 (41.5 - 52.8)	44 (28.5 - 45)	0.098[c]
Female, women, n (%)	13 (93)	22 (81)	0.097[a]
Disease duration	7.94 (2.38 - 11.9)	8.64 (5.03 - 15.6)	0.523[b]
AQP4-IgG positive, n (%)	11 (79)	16 (59)	0.457[a]
EDSS score (range)	3.0 (1.0 - 6.0)	2.0 (0 - 7.5)	0.267[b]
Number of relapses	3 (3 - 4)	2 (1 - 3.75)	0.093[b]
Current treatment, n (%)	13 (93)	24 (89)	0.999[a]
Beck Depression Inventory-Fast Screen (BDI-FS)	4.5 (0.7 - 8)	2 (0.5 - 5)	0.248[b]
Fatigue Impact Scale for Daily Use (D-FIS)	15.5 (5 - 19.8)	5 (2 -13)	0.109[b]
**Patient-centred measures**			
Physical MSIS-29v2	46.5 (31.8 - 58.8)	32 (23 - 43.5)	0.055[d]
Psychological MSIS-29v2	24.5 (17 - 32)	18 (11 - 26)	0.096[d]
Satisfaction with Life Scale (SWLS)	18 (15 - 21)	22 (20 - 27.5)	0.043[d]
SymptoMScreen questionnaire (SyMS)	26.5 (18.2 - 43.8)	11 (6 - 25.5)	0.043[d]
Stigma Scale for Chronic Illness (SSCI-8)	13.5 (10 - 16)	9 (8 - 11)	0.043[d]
MOS Pain Effects Scale (PES)	19 (12.5 - 21.8)	11 (8.5 - 19)	0.043[d]

The data represent the absolute numbers and proportions of qualitative data and the median and IQR for quantitative data, unless otherwise specified. CP: cognitively preserved; CI: cognitive impairment; AQP4-IgG: aquaporin-4 immunoglobulin G; EDSS: Expanded Disability Status Scale; MSIS-29v2: MultipleSclerosis Impact Scale.

[a]Chi-squared test; [b]Kruskal–Wallis test; [c]Student's *t*-test; [d]Age and sex-adjusted ANOVA and corrected by FDR adjustment.
